# Spectroscopic characterization of europium binding to a calmodulin-EF4 hand peptide–polymer conjugate†

**DOI:** 10.1039/d4ra01505c

**Published:** 2024-04-29

**Authors:** Dini Marlina, Yannic Müllers, Ulrich Glebe, Michael U. Kumke

**Affiliations:** a University of Potsdam, Institute of Chemistry, Optical Sensing and Spectroscopy Karl-Liebknecht-Str. 24–25 14476 Potsdam-Golm Germany kumke@uni-potsdam.de; b University of Potsdam, Institute of Chemistry, Polymer Materials and Polymer Technologies Karl-Liebknecht-Str. 24–25 14476 Potsdam-Golm Germany; c Fraunhofer Institute for Applied Polymer Research IAP Geiselbergstr. 69 14476 Potsdam-Golm Germany

## Abstract

The emergence of biological ligand as an alternative to chemical ligands enables a sustainable lanthanide extraction route. In this study, a peptide originating from the loop of domain 4 calmodulin (EF4) was synthesized and the interaction with europium ions was monitored using time resolved laser fluorescence spectroscopy (TRLFS). Despite being retracted from its full protein structure, the twelve amino acids of calmodulin-EF4 showed binding to europium. Europium-peptide complex formation was evident by an increase in decay time from 110 to 187 μs. The spectra of europium bound to peptide can be easily distinguished from the free europium ion as the ^5^D_0_ → ^7^F_2_ peak intensifies. When europium bound to the peptide–polymer conjugate, the decay time was further increased to 259 μs. This suggests that lanthanide binding can be enhanced by immobilizing the short peptide into a polymer matrix. The europium-peptide/conjugate bond was reversible, triggered by pH, promoting peptide reusability. Due to the fact that the study was conducted exclusively in water, it suggests minimal use of chemicals is possible while maintaining peptide affinity. This makes the calmodulin-EF4 peptide an ideal candidate as biological ligand. This study lays the groundwork for developing a peptide-based filter material for lanthanide separation.

## Abstract 2

A fundamental step in the development of peptide–polymer composites for novel lanthanide isolation modules using calmodulin-EF4 peptide as a binding motif for biological-motivated materials is presented.

## Graphical abstract

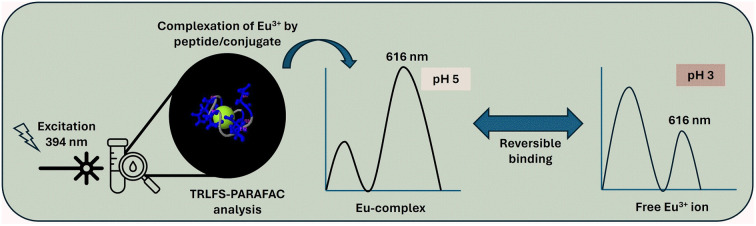

## Introduction

Lanthanides are enabling elements in contemporary technologies. As a consequence, the demand for lanthanides has increased rapidly along with technological developments, *e.g.*, related to renewable energy. The lanthanides are 4f elements starting from La (*Z* = 57) to Lu (*Z* = 71). The primary source of lanthanides are minerals, *e.g.*, monazite and bastnaesite. Lanthanides occur simultaneously in minerals, and the extraction as well as the separation from minerals are energy- and chemical–intensive processes.^1,2^ The challenges in lanthanides separation arise from the similarity of their physicochemical properties. In the minerals the +3 oxidation state prevails and the difference in ionic radii is extremely small, ranging from 1.2 Å for La^3+^ to ∼1.0 Å for Lu^3+^.^3^ This situation makes the tailoring of complexing agents specifically designed to recognize a particular lanthanide ion very challenging.^4^ As a result, the separation process needs to be repeated many times which releases large amounts of chemical waste that may cause water, soil, and air pollution.^5,6^ Moreover, mineral deposits also contain radioactive materials (Th, U), that if left unmitigated, can pose a severe risk to human health, for example, through inhaling contaminated dusts.^1^

According to a review conducted by McNulty *et al.*,^1^ the recoveries of lanthanides from their primary source are limited by current technology and plant practice to 50–80%.^1^ The increasing demand has caused the need to extract lanthanides from secondary sources such as acid mine drainage (AMD), coal and coal by-product, iron ore and apatite.^7^ Common complexing agents for lanthanides are, citrate, α-hydroxy-butyrate, or ethylenediaminetetraacetic acid (EDTA)^8^ In case of EDTA, it lacks in selectivity as it can bind divalent ions (Mg^2+^, Cu^2+^, Fe^2+^, Mn^2+^, Ni^2+^, and Zn^2+^).^9^ An alternative approach to overcome the lack of selectivity is to utilize specialized biological ligands.^10,11^ This way, the separation process can be trimmed reducing chemical as well as energy consumption being less harmful to the environment.

Biological ligands can be derived from proteins found in nature.^12–14^ Particular amino acid sequences located in calmodulin (CaM), a calcium ion (Ca^2+^) sensor protein, were found to form strong complexes with lanthanides. CaM is composed of 148 amino acids and binds Ca^2+^ in a putative helix-loop-helix domain known as the EF-Hand.^15^ The interactions of lanthanide ions with CaM have been known for decades. Due to the similar ionic radii, Ln^3+^ can replace Ca^2+^ and due to their unique optical properties (*e.g.*, Eu^3+^) can serve as a spectroscopic probe in CaM structure determination studies.^12,15–19^ Using the full protein as a potential ligand in a technical process might be difficult for various reasons. On the other side using a shorter amino acid sequence, which is directly involved in the metal binding is more favourable. CaM has four EF-Hands, labelled as EF1, EF2, EF3, and EF4, each consisting of 12 amino acids. The amino acid sequences of EF1–EF4 are almost identical, only differing in certain positions. The amino acid sequences of the EF-Hand motif are intriguing as a starting point in the course of finding efficient and selective novel ligands for lanthanide complexation.

Many metal binding affinity evaluations were carried out using the full CaM structure.^12,15–19^ CaM has a cooperative effect increasing the affinity for further Ln^3+^ ions after binding the first Ln^3+^ ion. Until now, most of the experiments have been carried out in a buffer solution, which is unlikely to be encountered in technical applications, *e.g.*, extraction of lanthanides from AMD. Some buffers (MOPS, MES, HEPES, and PIPES) at high concentration (>10 mM) were also found to interact with Eu^3+^ changing the speciation and because of the very similar chemical properties this can also be expected for the lanthanides in general.^20^ Consequently, buffers should be used with care to provide unbiased results that represent purely peptide–lanthanide interactions, which would be relevant in a remediation process for lanthanides from secondary sources.

When envisioning an effective and sustainable lanthanide separation system, the peptide should be functionalized or immobilized onto a solid support to enable an easy recovering and reuse of the peptide. Polymers are often used as solid support for the immobilization of biomolecules. In the search for a biomimetic peptide–polymer conjugate, a first but important step is to manage the coupling between both without eliminating the binding capabilities, but instead improving them due to a synergistic effect. This synergy is highly wanted since it is known from previous studies that short peptides lack the advantage of a pre-structuring due to the backbone in the full protein and subsequently show a low(er) binding affinity.

In this study as a first step to a biomimetic peptide-polymer-toolbox, we synthesized EF4 CaM peptide and its conjugate with the polymer poly(dimethyl acrylamide) (pDMA). Solid-phase peptide synthesis (SPPS) was exploited, being automatized, and enabling high purity and large scale especially for peptides with less than 50 amino acids.^21^ We investigated a model peptide for lanthanide binding based on the EF4 CaM, having the sequence DIDGDGQVNYEE. While still being immobilized on the resin for SPPS, a chain-transfer agent (CTA) for reversible addition–fragmentation chain-transfer (RAFT) polymerization was conjugated to the N-terminus of the peptide. After cleavage from the resin, pDMA was grown in a grafting-from approach.

The complexation of Eu^3+^ with peptide or peptide-polymer conjugate was investigated under mild acidic conditions (pH 5), without any buffer solutions, to simulate real-life wastewater conditions frequently found for mining drainage. It is advantageous to use pH 5 to avoid the formation of europium carbonates or hydroxides complexes since these complexes start to form at pH 7 and higher.^22^ Moreover, aspartic and glutamic acid, which are potential binding sites of peptides, are already deprotonated at this pH (with p*K*_a_ values of 3.9 and 4.2, respectively) and are subsequently fully available for cation binding.^23^ Complexation was monitored using time resolved laser fluorescence spectroscopy (TRLFS) and the luminescence spectra were deconvoluted using parallel factor analysis (PARAFAC) to identify the chemical species. The reversibility of binding between an Eu^3+^ ion and peptide was tested by lowering the solution pH and was monitored qualitatively using luminescence spectroscopy.

## Experimental

### Materials and reagents

*Tert*-butanoic acid butyl trithiocarbonate (BTMP) was synthesized according to a published procedure.^24^ DMA was purified over alumina to remove the inhibitors before polymerization. Millipore pure water with a resistivity of 18.2 MΩ cm was used. Europium was purchased as chloride salt (EuCl_3_·6H_2_O) from Sigma Aldrich and was used as received. It dissolved as a stock solution in deionized water and further diluted to desired concentration. A commercial EF4 Hand peptide (purity 95.9%) as a reference peptide was purchased from Peptide&Elephants.

### Peptide synthesis and characterization

#### Synthesis and grafting process

Peptides were synthesized through standard fluorenylmethoxycarbonyl (Fmoc)-based solid phase peptide synthesis. CTA is used as a linker to facilitate polymerization reaction. The details and scheme of the peptide synthesis, including the conjugation with the polymer pDMA, can be found on the ESI (Text S1†).

#### Peptide/peptide-polymer characterization

The characterisations were carried out using NMR spectroscopy, Reverse-Phase High-performance liquid chromatography (RP-HPLC), gel permeation chromatography (GPC), and electrospray ionization mass spectrometry (ESI-MS) (Text S2†).

### Binding studies of peptide-Eu^3+^

#### Titration of Eu^3+^ with peptide or peptide–polymer conjugate

The complexation of Eu^3+^ with peptide or peptide-polymer conjugate was monitored by TRLFS. The details on sample preparation for binding studies were provided in ESI (Text S3†). A series of titrations was carried out by adding peptide into the Eu^3+^ solution (1 or 10 μM) in cuvette increasing its total concentration. The decrease of Eu^3+^ luminescence intensity due to dilution during the titration process can be anticipated by adding the same amount of Eu^3+^ into the peptide stock solution used in the titration. In addition, the relative concentration of Eu^3+^ can also be adjusted during data analysis based on the knowledge of the added volume in each titration step.

Before luminescence measurement, the solution was mixed using a shaker (Vortex Genie 2, Scientific Industries) for at least 10 s. The measurements were carried out at room temperature. Eu^3+^ was excited at *λ*_ex_ = 394 nm with a pulsed Nd:YAG laser (Quanta Ray, Spectra Physics, with repetition rate 20 Hz or 10 Hz). The luminescence was monitored at 570 nm < *λ*_em_ < 720 nm. The detector used was an iCCD camera coupled to a spectrograph (iStar DH734-18H-13, Andor technology coupled to Shamrock 303i-A, SR0275, Andor technology for the 20 Hz laser or iStar sCMOS, Andor technology coupled to Kymera328i, Andor technology for the 10 Hz laser). In both detection systems a grating with 300 lines per mm with *λ*_blaze_ = 760 nm (20 Hz laser) or 500 nm (10 Hz laser) was used.

The boxcar technique was used to measure time resolved emission spectra and subsequently to obtain the emission kinetics. The slit size, gate width, initial delay time, and gate delay step were adjusted according to the laser used (Table S1†). The luminescence spectra were corrected for the spectral sensitivity of the grating and of the iCCD camera, respectively.

#### Speciation analysis using PARAFAC

**Fig. 1:**
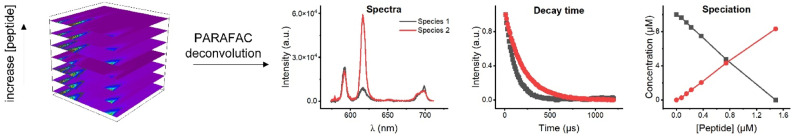
Four-dimensional data cube in PARAFAC analysis consists of emission wavelength, intensity, and time as *x*-, *y*-, *z*- axis, respectively, with different peptide concentration as the fourth axis. PARAFAC deconvolution yielded information regarding the species.

The 3-dimensional TRLFS data consist of emission wavelength as *x*-axis, intensity as *y*-axis, and time in *z*-axis. Using PARAFAC for the data analysis, all TRLFS data from different peptide concentration were combined yielding the fourth dimension (Fig. 1). Some constraints were used in the analysis, such as, monoexponentially constraint in the time dimension, and non-negative (for speciation and luminescence spectra). As a result of PARAFAC analysis, information on amount of each Eu^3+^ species as well as the species-selective luminescence spectra and respective decay times are obtained. The PARAFAC analysis was run using MATLAB2022b.

In the luminescence measurements the data were collected for the spectral range between 570 nm < *λ*_em_ < 720 nm, covering the transitions up to ^5^D_0_ → ^7^F_4_. Using the species-selective emission spectra obtained from the PARAFAC analysis the luminescence quantum efficiencies of the different species were calculated based on the Judd–Ofelt theory using an in-lab written routine for MATLAB 2022b. The quantum efficiencies (*ϕ*) of the different Eu^3+^ species in the solutions were used to determine the concentrations and subsequently the dissociation constant (*K*_D_) values. The relative concentration of each species (*C*_*i*_) was calculated from its luminescence intensity (*I*_*i*_) and the quantum yield (*ϕ*_*i*_) according to the eqn (1):^25^1
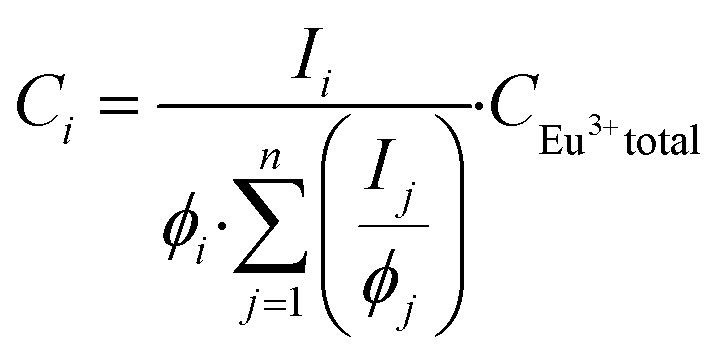


To connect the photophysical data in a more intuitive way to the Eu^3+^-complexes, the fluorescence decay time was used. The estimation of the number of water molecules (*n*) in the first coordination sphere of Eu^3+^ was calculated based on eqn (2):^15^2
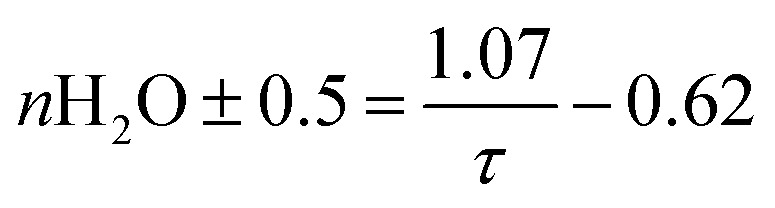


## Results and discussion

### Peptide and peptide-polymer characteristic

The EF4 peptide was synthesized in a standard SPPS approach on an automated peptide synthesizer.^21^ A resin with 2-chlorotrityl (2-CT) linker and preloaded with glycine was exploited for SPPS, resulting in DIDGDGQVNYEEG (EF4G) peptide (Scheme S1†). Subsequently, while still being immobilized on the resin, the peptide was functionalized with a CTA for RAFT polymerization. As SPPS takes place from the C- to the N-terminus, the CTA *tert*-butanoic acid butyl trithiocarbonate (BTMP) with carboxylic acid functionality was chosen to be linked to the peptide's N-terminus *via* the same amide bond forming chemistry as the amino acids before. In addition, the trithiocarbonate structure of the CTA is more stable towards amines than other CTAs.^26^ The aim of our study was to investigate the influence of polymers that are covalently connected to the peptide on the Ln binding properties. To synthesize peptide–polymer conjugates, we chose RAFT polymerization which is a frequently used controlled radical polymerization. For RAFT, a CTA is required to mediate the polymerization process. In this reaction, the peptide species (R-group) functions as a radical, while the long alkyl chain in the CTA (Z-group) mediates the stability of the S=C bond and the adduct radical. Theoretically, it would be possible to connect peptide and CTA *via* the Z-group, however this would be synthetically more complicated with unknown influence on polymerization.

After cleavage from the resin and precipitation in cold diethylether, ESI mass spectrometry confirmed the expected masses (Fig. S1†). HPLC analysis demonstrated a sufficient purity of the peptide (Fig. S2†). As shown in Fig. S3,† the conjugation of BTMP to the peptide resulted in a mixture of unmodified peptide and CTA-peptide. This was probably caused by a non-quantitative coupling reaction due to high steric hindrance of the carboxylic acid group of BTMP through both alpha-methyl groups. The purity of the conjugate was determined to be 52% and was calculated by the ratio of absorbance at 220 nm of modified and unmodified peptide. Since EF4G does not interfere in the polymerization reaction, the CTA-peptide was used for polymerization without further purification. Such a grafting-from approach is advantageous regarding ease of purification and ensures that nearly all polymers carry a peptide end group.^27^ DMA was polymerized with a targeted degree of polymerization of 160. Monomer conversion was 96% as determined by NMR spectroscopy of the crude polymer mixture. GPC analysis of pDMA-EF4G (Fig. S4†) showed a well-controlled reaction with a product dispersity *Ð* of 1.3. The number weighted molar mass Mn was determined to 17.6 kDa which fits well with the theoretical mass of 17.2 kDa calculated through monomer conversion. ^1^H-NMR spectroscopy of both peptide and peptide-polymer conjugate (Fig. S5†) show the characteristic proton signals of the amide backbone as well as asparagine and glutamine sidechains in the low field of the spectrum. This indicates the successful implementation of EF4G into the polymer.

### Luminescence spectroscopy studies

The Eu^3+^ luminescence originates from the ^5^D_0_ excited state to the ^7^F_J_ (*J* = 0 to 6) ground states and the spectral intensity distribution of the Eu^3+^ luminescence is very sensitive to the actual ligand field. Therefore, Eu^3+^ was used as the luminescence probe of choice to establish a procedure for the investigation of novel polymer–peptide conjugates as binders for the extraction of lanthanides. However, since the ^5^D_0_ → ^7^F_J_ (*J* = 5,6) transitions are in the NIR spectral range and usually low in intensity, in this study the luminescence spectra were monitored only in the wavelength range of 570 nm < *λ*_em_ < 720 nm, covering the transitions from ^5^D_0_ → ^7^F_J_ (*J* = 0 to 4), which is sufficient to apply Judd–Ofelt theory for the further calculation of specific photophysical parameters such as the luminescence quantum yield (*vide infra*).

**Fig. 2:**
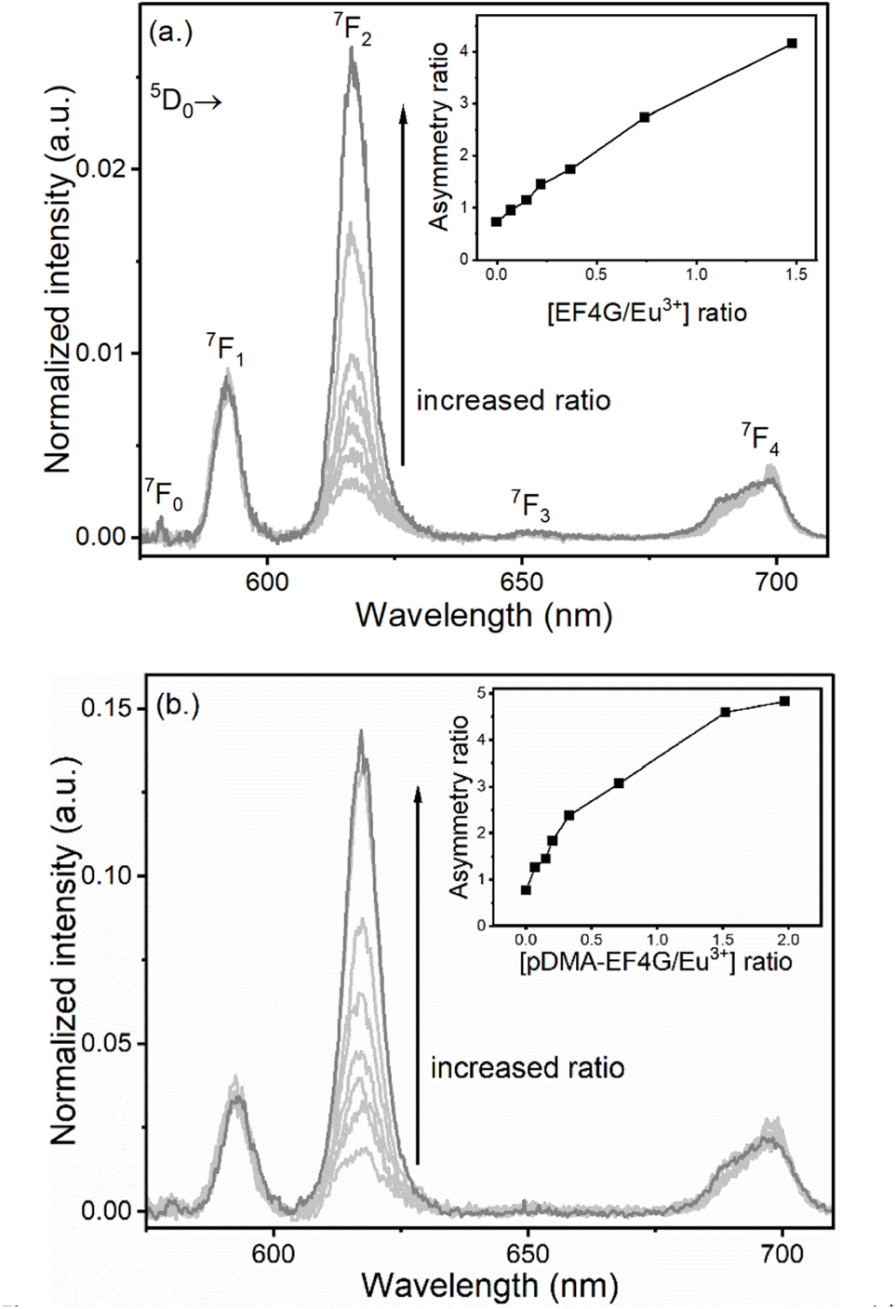
Luminescence spectra of Eu^3+^ at pH 5 taken at gate delay 10 μs with increasing concentration of (a) EF4G and (b) pDMA-EF4G. The complexation of Eu^3+^ with the peptide indicated by the increase of the ^5^D_0_ → ^7^F_2_, “the hypersensitive” peak intensity; inset: asymmetry ratio.

In Fig. 2 the alterations of the Eu^3+^ luminescence spectrum upon addition of EF4G or pDMA-EF4G are shown. In the absence of EF4G (or pDMA-EF4G), at pH 5 Eu^3+^ exists as an aquo ion characterized by the dominance of the ^5^D_0_ → ^7^F_1_ transition peak. Because in the aquo ion the coordination sphere of the Eu^3+^ ion with 8 to 9 water molecules is highly symmetric, the ^5^D_0_ → ^7^F_0_ transition is strictly forbidden and not seen in the emission spectrum at RT.^28^ Meanwhile, for the Eu^3+^ peptide complexes (EF4G as well as pDMA-EF4G), the spectral intensity distribution of the Eu^3+^ emission is distinctly changed. In contrast to the aquo ion now the luminescence spectra are dominated by the ^5^D_0_ → ^7^F_2_ transition peak (*λ*_em_ = 616 nm). This transition is often indicated as “hypersensitive”, which means that its intensity is strongly influenced by the local symmetry of the Eu^3+^ ion and the nature of the ligand. In combination with the ^5^D_0_ → ^7^F_1_ transition (magnetic transition, which is not affected by the ligand field and therefore useful as internal standard), the intensity ratio of (^5^D_0_ → ^7^F_2_/^5^D_0_ → ^7^F_1_) peaks (asymmetry ratio) is often used as an indicator of alterations in the Eu^3+^ coordination site.^29^ It is worth mentioning that asymmetry ratios can already be extracted from the raw data without advanced data treatments to visualize the progress of the complexation upon addition of peptide (inset Fig. 2a and b). The asymmetry ratio of the respective complex can be extracted from the species-selective spectra obtained from PARAFAC analysis (*vide infra*, Table 1).

**Table 1:** Summary of characteristic of each Eu^3+^ species deconvoluted using PARAFAC

	Eu^3+^ aquo ion	Eu^3+^-EF4 (ref.)	Eu^3+^-EF4G	Eu^3+^-pDMA-EF4G
^5^D_0_ → ^7^F_0_ peak position (nm)	—	579.37	579.15	579.9
Asymmetry ratio	0.8	3.5	3.7	4.9
Decay time (*τ*) in μs	110 ± 10^a^	163 ± 1	187 ± 1	259 ± 2
Number of water molecules (*n*)	9 ± 0.5	6 ± 0.5	5 ± 0.5	3.5 ± 0.5
Quantum yield (*φ*) in %	1 ± 0.2	3.2 ± 0.2	3.8 ± 0.2	7.1 ± 0.2
JO parameter				
(Ω2) (10^−20^ cm^2^)	1.3	6.2	6.7	9.0
(Ω4) (10^−20^ cm^2^)	2.7	2.8	2.6	4.4
Dissociation constant (*K*_D_) in μM^b^	—	8.9	8.0	0.4

a Data averaged from three experiments.

b Standard deviation of *K*_d_ was estimated to be 50% from the stated value.

Another direct indication for the complexation of Eu^3+^ by the peptides is the appearance of the ^5^D_0_ → ^7^F_0_ peak (see Table 1 for *λ*_em_). For the Eu^3+^ complex with EF4G the ^5^D_0_ → ^7^F_0_ peak was more prominent meanwhile for the pDMA-EF4G conjugate the ^5^D_0_ → ^7^F_0_ peak was weaker and monitoring the complexation based on this particular parameter would be challenging.

In addition, also the ^5^D_0_ → ^7^F_4_ spectral distribution and relative intensity was slightly changing upon complexation with EF4G or pDMA-EF4G (see Fig. 2). In general, the intensity of this band is weaker than the other luminescence peaks in the Eu^3+^ luminescence spectra.^29^

Based on the raw luminescence data presented in Fig. 2, it can already be concluded that EF4G as well as pDMA-EF4G are complexing Eu^3+^ in an aqueous solution at pH 5, which is lower than the physiological pH under which peptides or the full calmodulin normally would be tested.^12,16^ Moreover, based solely on the alteration of the spectral intensity distribution (asymmetry ratio, intensity of luminescence peaks), which was found to be similar but not identical for both ligands (EF4G *vs.* its polymer conjugate). From the distinct differences an influence of the polymer on the overall binding is indicated.

As a comparison, we also tested the affinity of a commercially obtained EF4 (“only” 12 amino acids) and compared to our EF4G (13 amino acids) we found identical binding characteristics. This shows that the additional glycine added to the EF4 sequence does not influence the peptide binding (see Table 1 and Fig. S6†). Additionally, the potential contribution to Eu^3+^ binding from the polymer itself was checked by adding free polymer (pDMA) only. Here, no alteration in the spectral intensity distribution of the Eu^3+^ luminescence was found indicating that, in absence of peptide, the polymer does not complex the Eu^3+^ ion (see Fig. S7†).

Although the increased binding by EF4G (or pDMA-EF4G) can be qualitatively shown by the dependence of the asymmetry ratio and partly, by the intensity increase of the ^5^D_0_ → ^7^F_0_ transition, the determination of the dissociation constant (*K*_D_) values to quantify the binding based on the raw data is not straightforward since either the luminescence signal is a sum of contributions from the aquo ion and the Eu^3+^ bound to the peptide (*e.g.*, affects the absolute value of the asymmetry ratio) or the overall intensity of the ^5^D_0_ → ^7^F_0_ transition is small and therefore not very precise. In order to determine the *K*_D_, the concentrations of aquo ion as well as of the peptide complex needs to be known at each step of the titration. Therefore, the raw data have to be deconvoluted into the species related fractions. This was carried out applying a PARAFAC analysis in combination with Judd–Ofelt theory to the experimental TRLFS data (*vide infra*), because for a quantitative analysis of the luminescence data the luminescence quantum yields of the species is needed.

### TRLFS data analysis

TRLFS titration data of Eu^3+^ with EF4G and pDMA-EF4G were deconvoluted using parallel factor analysis (PARAFAC). PARAFAC is a routine analysis used to deconvolute multidimensional emission spectra.^25,30–36^ In the PARAFAC analysis, fitting the data with a two-species-model yielded for both ligands the best result.

**Fig. 3:**
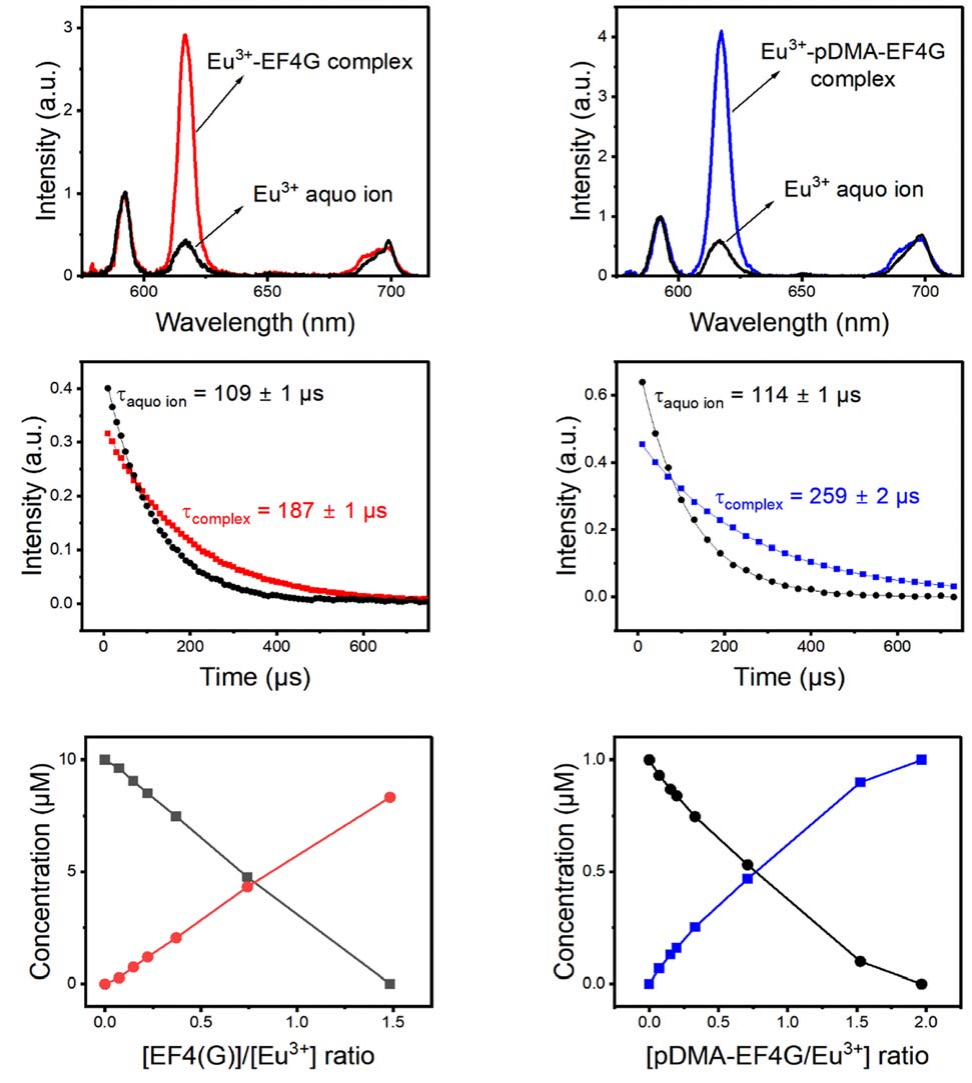
PARAFAC deconvolution of TRLFS of Eu^3+^ complexation with EF4G and pDMA-EF4G. From top to bottom: spectrum of each species, decay time, and species distribution.

For the investigated molar ratios between Eu^3+^ and the ligands no indication of a further species was found and therefore the two species were attributed to the Eu^3+^ aquo ion and the 1 : 1 Eu^3+^-EF4G complex (or the Eu^3+^-pDMA-EF4G complex). The obtained species-selective luminescence spectra, the respective luminescence decay kinetics (and luminescence decay times) as well as the relative fractions of the species at different molar ratios of Eu^3+^ and ligand are shown in Fig. 3.

The decay time (*τ*) of Eu^3+^ aquo ion is found to match with literature data.^15,22^ A *τ* of 110 ± 10 μs was determined for the aquo ion for all ligand systems (including controls) and based on eqn (2) this corresponds to 8–9 water molecules in the first coordination sphere. The decay time of Eu^3+^ bound to EF4G is determined to *τ* = 187 ± 1 μs, which corresponds to an average of 5 ± 0.5 water molecules. This decay time is distinctly shorter in comparison with EF4 in an intact calmodulin (*τ* = 405 ± 2.0 μs) which indicates that the binding situation using a short peptide is very different due to the missing pre-structuring and additional binding by the protein's backbone.^15^ In a short water-soluble peptide, the interactions between hydrophobic side chain and hydrophilic backbone segments are interspersed, precluding the formation of specific intramolecular hydrophobic clusters. In consequence, the short linear peptides would adopt random structures in water solution.^37^ This behaviour is confirmed by a report on isolated EF3 calmodulin that was found to be in a random configuration.^19^ As EF3 and EF4 calmodulin sequences differ only slightly, we assume that this random structure applies to isolated EF4 calmodulin as well.

Considering the larger average number of water molecules in the first coordination sphere (5 for EF4G *vs.* 2 for the full protein, calculated from the luminescence decay time using eqn (2)), a multi-dentate binding can be ruled out (indication of only two water molecules being removed from first coordination sphere of aquo ion), which means the peptide seems to bind only *via* bidentate motif.

The conjugation with the polymer pDMA increased the decay time of the Eu^3+^-complex to *τ* = 259 μs corresponding to an average of 3–4 water molecules in the first coordination. This implicates that more binding sites from the pDMA-EF4G are attached to the Eu^3+^ ion. The attachment of the peptide to polymer enhances the rearrangement of the binding sites, improving the folding structure of the peptide. A similar effect was observed in the complexation of europium with 33-mer peptides that bind to DNA. There is a decrease in the number of water molecules in the first coordination sphere of europium, which results in a longer decay time.^38^

The complexes Eu^3+^-EF4G and Eu^3+^-pDMA-EF4G have asymmetry ratios of 3.7 and 4.9, respectively. According to literature data, this finding is consistent with the ratio of Eu^3+^ bound to sites 2–4 in calmodulin, which has a ratio of 4.^15^ From the fact that the observed asymmetry ratios are all very similar for the different complexes, it is shown that this parameter is more sensitive to the overall symmetry instead of the nature of the ligands. Here, at least in an aqueous milieu the luminescence decay time is the stronger indicator.

### Influence of pH on the complex formation

**Fig. 4:**
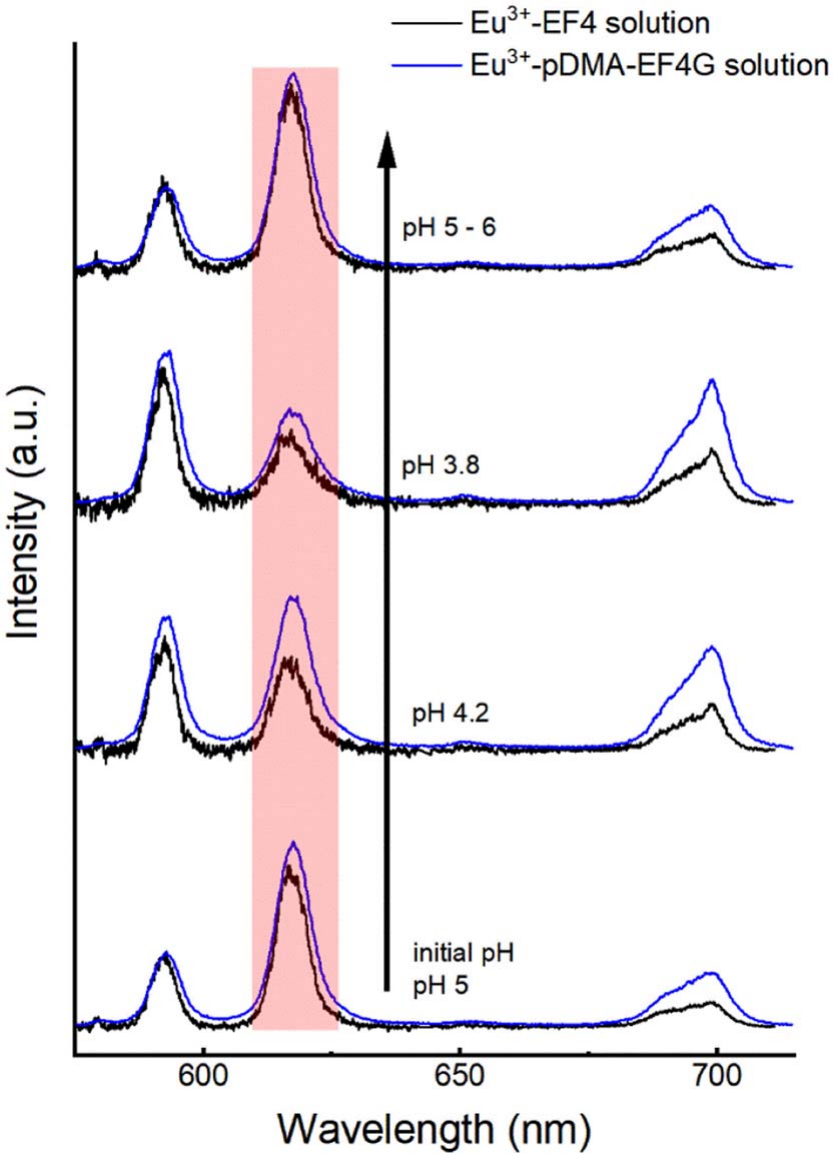
Spectral change of Eu^3+^-EF4/pDMA-EF4G (1 : 1 molar ratio) luminescence upon alteration of the pH. The change in intensity of ^5^D_0_ → ^7^F_2_ (hypersensitive) peak indicates the binding reversibility: at pH 4.2 the intensity started to decrease, at pH 3.8 the luminescence spectrum resembles largely the Eu^3+^ aquo ion upon increasing the pH back above 5 the luminescence of the peptide complex is observed again.

**Fig. 5:**
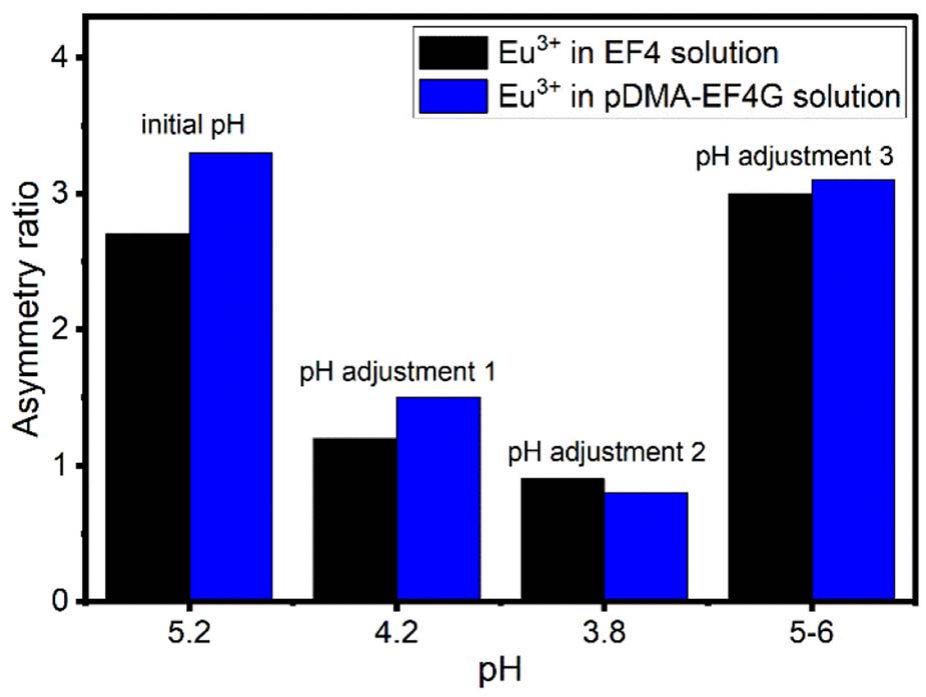
The change in asymmetry ratio of Eu^3+^ in EF4/pDMA-EF4G solution at different pH. Black: Eu^3+^ in EF4 solution, blue: Eu^3+^ in pDMA-EF4G solution. Asymmetry ratio < 1 indicates the free Eu^3+^ aquo ion.

The reversibility of the Eu^3+^ binding to the peptide material is of high importance for further potential technical applications. We have investigated the pH as a potential trigger to release the Eu^3+^ from the polymer-peptide complexes. A variation of the pH in the range of 3 < pH < 6 was tested, which on the one hand side can be expected for natural water (*e.g.*, from mining drainage) and on the other hand should not cleave the peptide. The Eu^3+^-peptide-polymer (at a molar ratio 1 : 1) binding was qualitatively monitored by observing the changes in the Eu^3+^ luminescence spectrum (Fig. 4). At the initial pH, the spectrum is characteristic for the Eu^3+^: EF4G/pDMA-EF4G complex. As the pH is decreased to around 3.8, the spectrum now becomes characteristic for the aquo Eu^3+^ ion indicating a release of the Eu^3+^ from the peptide complex. Re-binding of Eu^3+^ is achieved when the pH is adjusted back to 5–6. The asymmetry ratio (Fig. 5) points to different Eu^3+^ species according to the solution pH. At pH 5–6 the asymmetry ratio is ≥3 indicating Eu^3+^ binding to the peptide and at pH ∼3, the asymmetry ratio is <1, characteristic for Eu^3+^ aquo ion. As isolated EF Hand has no particular conformational constraints in aqueous solution, the binding process is governed by the functional group present in the side chains. According to the literature, the coordination positions of EF Hand in intact protein are located at position 1, 3, 5, 7, 9 and 12.^39^ In the case of isolated EF4 CaM, not all amino acids at those positions bind to Eu^3+^. Since the binding reversibility is highly affected by the pH, it is likely that the acidic side chain of aspartic and glutamic acid play role in the coordination with Eu^3+^. The binding reversibility of lanthanide ions with bioinspired ligands has also been observed with Lanmodulin.^40,41^

## Conclusions

The utilization of peptides as biological ligands for lanthanide complexation was demonstrated for a small peptide fragment of the metal-binding site of calmodulin and Eu^3+^ as a representative of lanthanide elements. First, the peptide EF4G was synthesized using the SPPS method and showed in control experiments binding of Eu^3+^. In the next step a chain-transfer agent (CTA) for RAFT polymerization was conjugated to the N-terminus of the peptide, which was still immobilized on the SPPS resin. With this precursor material an important step for the further synthesis of peptide–polymer conjugates was made. In a first prove of concept, pDMA was grown in a grafting-from approach to obtain the pDMA-EF4G conjugate which was further characterized for its Eu^3+^ binding properties.

Conjugation of the peptide to the polymer pDMA has a synergistic effect on the Eu^3+^ binding as can be evidenced from TRLFS-PARAFAC analysis. The increased decay time (compared to EF4G) of the Eu^3+^-conjugate complex is caused by the exclusion of more water from the Eu^3+^ first coordination sphere. It is attractive to assume that the polymer improves the binding of Eu^3+^ by pre-structuring the peptide sequence (like the contribution of the backbone in the full protein) making a multidentate binding more favourable even for such a small molecule. In order to be used in the recycling process of lanthanide ions, in addition to high affinity and high selectivity, the reversibility of the binding process to the peptide material is of high importance. Hence, a key requirement for the envisioned biomimetic lanthanide binding polymer–peptide conjugates has to be the reversibility of Ln binding to such ligands. For the pDMA-EF4G conjugate the release of Eu^3+^ was easily achieved by changing the pH of the solution. This feature is very promising for the future development of bio-based resins for lanthanide extraction.

Future research will focus on varying amino acid sequence of the peptide, the polymer type and length to optimize the binding with respect to strength but also for selectivity towards different lanthanides ions. Especially stimuli-responsive polymers seem to be very attractive to tailor the properties for ion binding and release. Through the use of thermoresponsive polymers such as poly(*N*-isopropylacrylamide), a thermal release of ions could be envisioned.

With the successful synthesis of the pDMA-EF4G conjugate a blueprint for a novel class of peptide-based filter materials for lanthanide separation is on hand. In our future work we will extend this approach towards a toolbox with different polymers available for conjugation. Work is in progress to tackle the other equally important tasks which are the investigation of the selectivity towards different lanthanides and the competition with other metal ions (*e.g.*, alkali metals, d-metals) most likely present in real-world samples.

## Conflicts of interest

There are no conflicts to declare.

## Supplementary Material

RA-014-D4RA01505C-s001
