# Supplementary material for: Spectroscopic characterization of europium binding to a calmodulin-EF4 hand peptide–polymer conjugate
Source: RSC Adv. 2024 Apr 29;14(20):14091–9. doi: 10.1039/d4ra01505c (PMC11056824; doi:10.1039/d4ra01505c)
Supplement: RA-014-D4RA01505C-s001 [file RA-014-D4RA01505C-s001.pdf]

Electronic Supplementary Material (ESI) for RSC Advances.
This journal is © The Royal Society of Chemistry 2024

Supporting Information

Spectroscopic characterization of europium binding to a calmodulin-EF4 hand peptide-polymer conjugate

Dini Marlina[a], Yannic Müller[b,c], Ulrich Glebe[b,c], and Michael U. Kumke*[a]

a. University of Potsdam, Institute of Chemistry, Optical Sensing and Spectroscopy, Karl-Liebknecht-Str. 24–25, 14476 Potsdam-Golm, Germany

b. University of Potsdam, Institute of Chemistry, Polymer Materials and Polymer Technologies, Karl-Liebknecht-Str. 24–25, 14476 Potsdam-Golm, Germany

c. Fraunhofer Institute for Applied Polymer Research IAP, Geiselbergstr. 69, 14476 Potsdam-Golm, Germany.

*Corresponding author:

apl. Prof. Dr. Michael Kumke, Email address: kumke@uni-potsdam.de

Text S1. Peptide synthesis:

Peptides were synthesized through standard fluorenylmethoxycarbonyl (Fmoc)-based solid phase peptide synthesis with glycine preloaded 2-Chlorotrityl resin (0.79 mmol g-1) on an Intavis MultiPep RSi automatic synthesizer. A typical procedure consisted of 100 µmol resin with cleavage of the N-terminal Fmoc protection group through a mixture of 20% piperidine and 5% formic acid in N,N-dimethylformamid (DMF) (1 x 3 min, 1 x 17 min). For the coupling of Fmoc-protected amino acids, 3.9 eq. 2-(1H-benzotriazole-1-yl)-1,1,3,3-tetramethyluronium hexafluorophosphate (HBTU), 4 eq. amino acid and 8 eq. N-methylmorpholine (NMM) were used (1 x 60 min). Peptide-CTA conjugates were prepared by amide coupling of 2 eq. BTMP with 1.9 eq. HBTU and 4 eq. NMM (2 x 60 min). After each coupling step, the resin was treated with 0.85 eq. acetic anhydride in DMF (1 x 30 min). Peptides were cleaved off using a mixture of trifluoroacetic acid (TFA)/ triisopropyl silane (TIPS)/water (95:2.5:2.5) for 2 hours and isolated by precipitation in cold diethyl ether. The purity of EF4G and CTA-EF4G was determined by reverse phase HPLC. The obtained products were verified by ESI-MS and 1H-NMR spectroscopy. EF4G and CTA-EF4G were used without further purification.

Grafting-from polymerization

10 mg CTA-EF4G (6.1 µmol, 1 eq.), 96 mg DMA (0.97 mmol, 160 eq.) and 0.6 mg azobis(isobutyronitrile) (AIBN) (0.61 µmol, 0.1 eq.) were solubilized in 500 µl dimethyl sulfoxide (DMSO) and placed in a Schlenk flask. The reaction mixture was degassed through 3 freeze-pump-thaw cycles and subsequently placed in a pre-heated oil bath at 65°C. After 16 hours, the reaction was stopped through the introduction of air into the reaction vessel. The mixture was diluted with approx. 4 ml water and dialyzed for 7 days against Milli-Q water, during which the solvent was changed 5 times. In order to ensure removal of residual peptide and monomer, a dialysis tube based on regenerated cellulose (Sigma Aldrich, MWCO 14.4 kDa) was used. The solvent was then removed by freeze-drying and the conjugate analyzed via 1H-NMR spectroscopy and GPC.

Text S2. Peptide/peptide-polymer conjugate characterization

NMR Spectroscopy

^{1}H NMR spectra were recorded using a Bruker Avance 300 NMR spectrometer. Chemical shifts δ are given in ppm referring to the respective solvent peak at δ 2.50 ppm for DMSO.

Reverse-Phase High-Performance Liquid Chromatography (RP-HPLC)

Measurements were performed using an Agilent 1200 system (Agilent Technologies Inc.), equipped with a degasser, autosampler, multi-wavelength DAD detector and automated fraction collector. The column used for analytical runs was a Brownlee SPP2.7 (C18, 2.1x150 mm) from PerkinElmer. The solvent system was an aqueous sodium phosphate buffer (20 mM, pH 7) and methanol (MeOH) with a flow rate of 0.2

mL/min. Solvent purity was HPLC gradient grade or higher. For instrument control and data collection, ChemStation B03.02 (Agilent Technolgies, Inc.) was used. Peak analysis and baseline correction were done using Origin Pro 9.1. Purity calculations were done using the absorption at short-wavelength UV (220 nm).
Method A for EF4G: 5-20 % MeOH within 30 min, linear gradient, isocratic 70 % MeOH for 10 min, 5 % MeOH for 15 min for re-equilibration.
Method B for CTA-EF4G: 5-70 % MeOH within 30 min, linear gradient, isocratic 70 % MeOH for 10 min, 5 % MeOH for 15 min for re-equilibration.

Gel Permeation Chromatography (GPC)

For GPC analysis, N-methyl-2-pyrrolidon (NMP) was used as the eluent, with the addition of LiBr (0.1%) as an eluent modifier. A flow rate of 0.5 mL/min was used with a high-pressure liquid chromatography pump (Spectra Systems Materials P 1000) and a refractometer detector SEC-3010 (WGE Dr. Bures). Calibration was performed using a polystyrene standard, and the results were evaluated using the PSS WinGPC UniChrom software.

Electrospray Ionization Mass Spectrometry (ESI-MS)

Spectra were recorded using a single quadrupole Flexar SQ 300 MS detector (PerkinElmer). The instrument was controlled using SingleQuad 2.2. Before measurement, the sample was dissolved in MS grade ammonium acetate (10mM, pH7). All spectra were recorded in negative ion mode.

Text S3. Sample preparation for binding studies of peptide-Eu^{3+}

The europium solution (1 or 10 µM) was prepared by diluting the stock solution (0.07 M). Peptide and polymer-peptide solutions were prepared by diluting approximately 0.5 – 1 mg mL-1 in MilliQ water. The solution pH was adjusted to pH around 5 by adding sodium hydroxide (NaOH) or perchloric acid ($HClO_4$) solutions and the pH was measured using a pH meter (inoLab pH 720). The concentration of the peptide/peptide-polymer was measured in 1 cm PMMA cuvette using UV-Visible spectrometer (Perkin Elmer). The concentration was calculated based on absorption at 280 nm and the extinction coefficient of tyrosine (ε) 1490 M-1 cm-1.

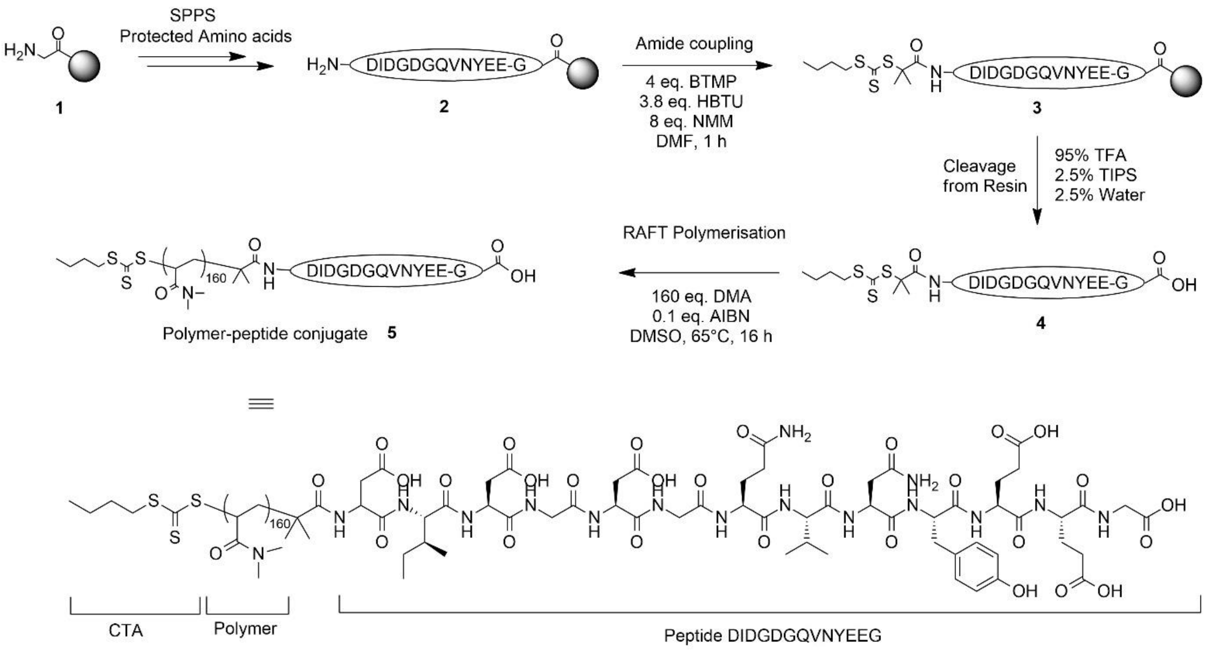

Scheme S1. Synthesis of polymer-peptide conjugate: SPPS with protected amino acids on a preloaded 2-CT resin 1 yields immobilized EF4 peptide 2. In the next step, BTMP is conjugated via amide coupling resulting in 3. The product 4 is cleaved from the resin and used in thermal RAFT polymerisation with 160 eq. DMA giving the polymer-peptide conjugate 5.

Table S1 Parameter details for TRLFS measurements

**Parameter**	**Eu^{3+}-EF4G**	**Eu^{3+}-pDMA-EF4G**
**Laser frequency (Hz)**	20	10
**Number of accumulations**	100	100
**Number in kinetic series**	120	25
**Gain level**	100	2500
**Gate width (ms)**	1	1
**Gate delay (µs)**	10	10
**Gate delay step (µs)**	10	30
**Slit size (µm)**	100	300

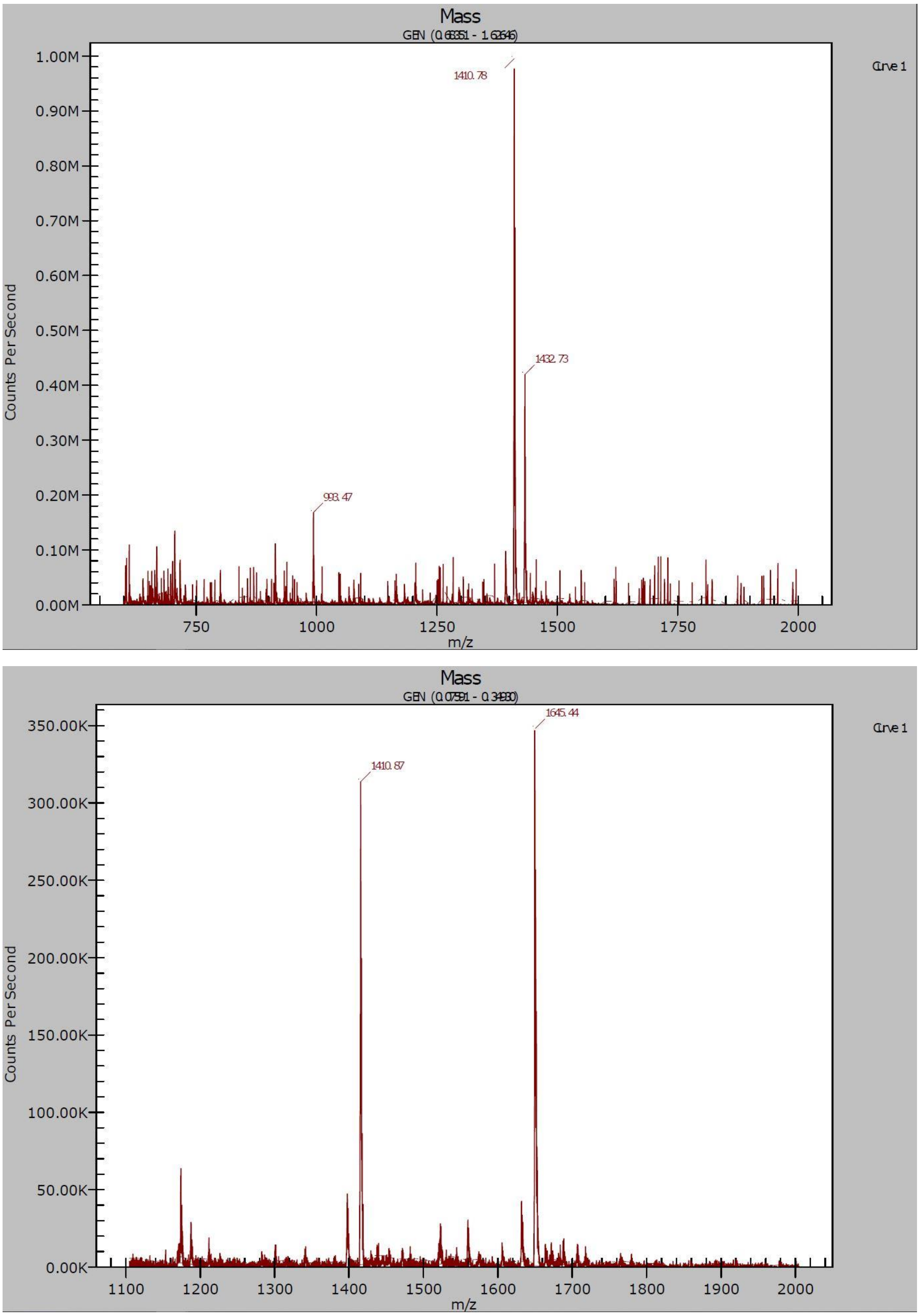

Figure S1 ESI mass spectra of EF4G (top) and CTA-EF4G (bottom).

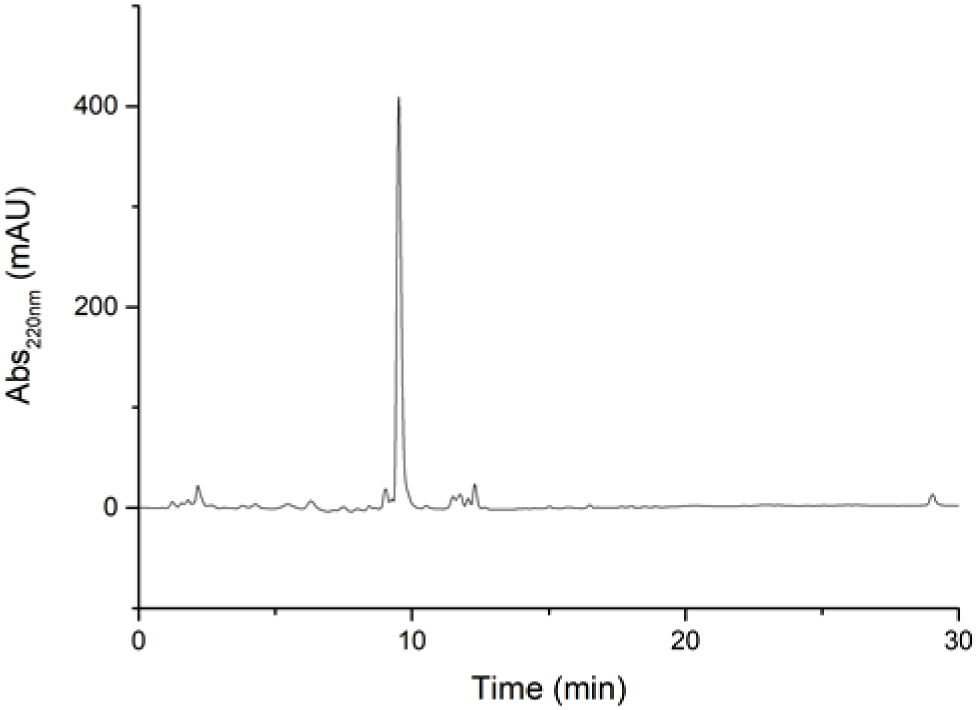

Figure S2 HPLC chromatogram of EF4G using method A.

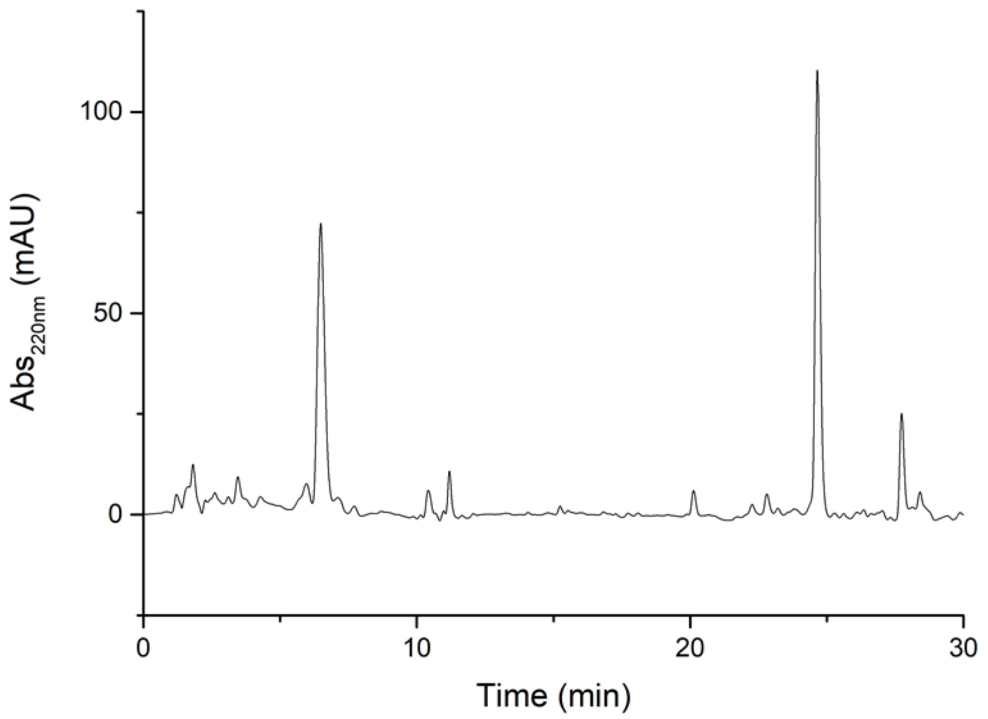

Figure S3 HPLC chromatogram of CTA-EF4G using method B.

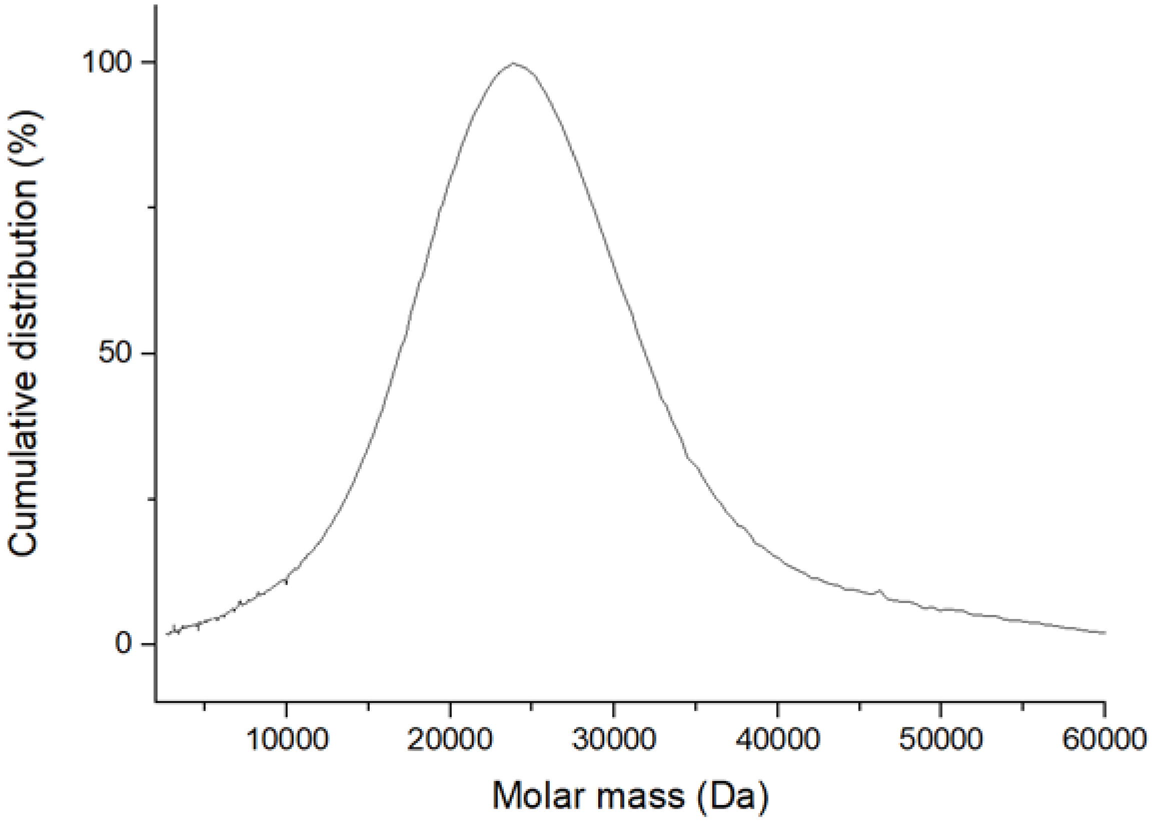

Figure S4 Molar mass distribution of pDMA-EF4G determined by GPC.

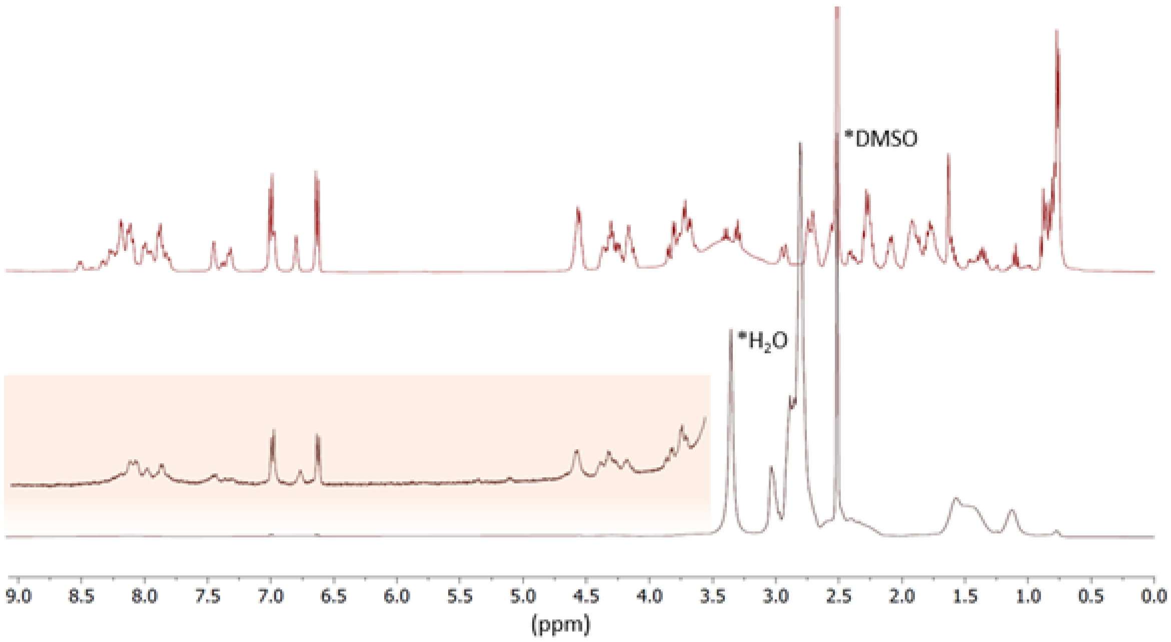

Figure S5 ^{1}H-NMR spectra of EF4G (top) and pDMA-EF4G (bottom) in d_6-DMSO. Insert is a zoom-in of the signals between 3.5 and 9.0 ppm.

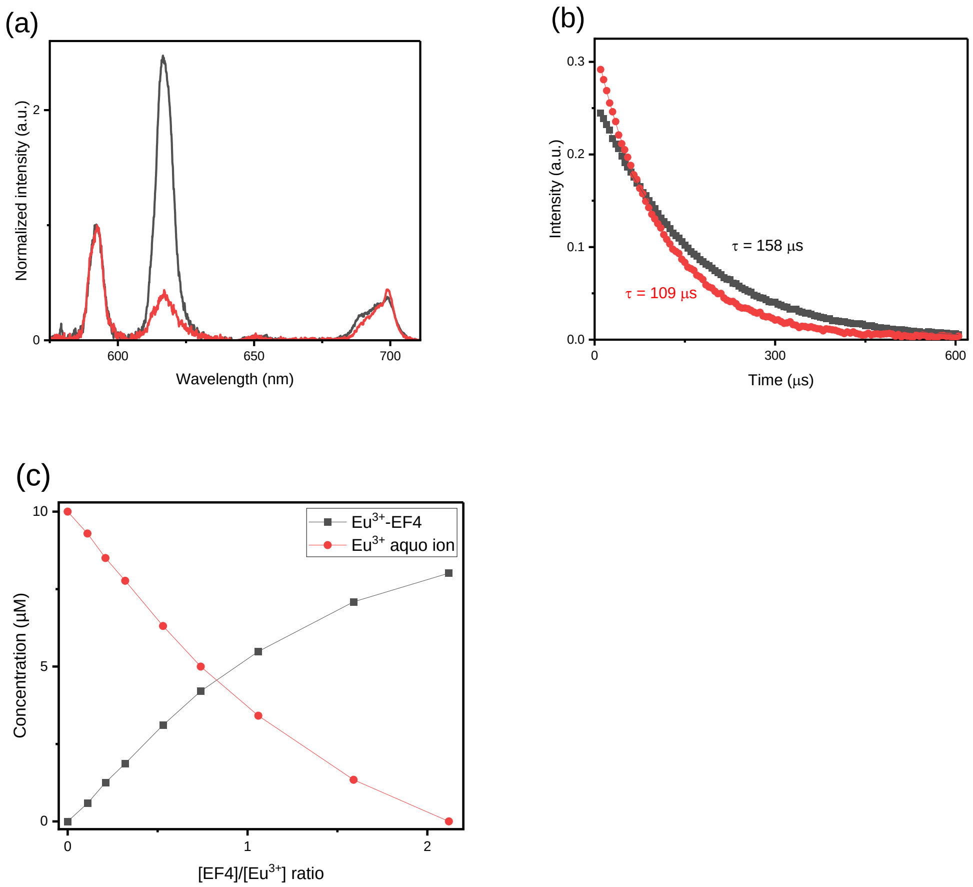

Figure S6 PARAFAC deconvolution of titration of Eu^{3+} with commercial EF4 peptide: a) luminescence spectra, b) decay time, c) speciation diagram. (color index: red = Eu^{3+} aquo ion, black = Eu^{3+}-EF4 complex).

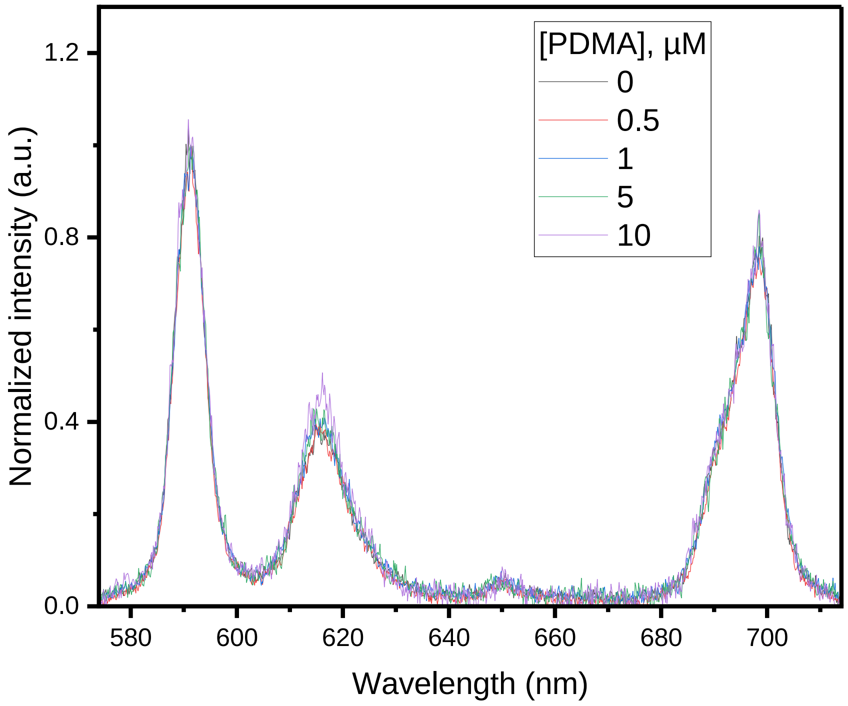

Figure S7 Luminescence spectra of Eu^{3+} 1 µM (λ_{ex} = 394 nm) in the presence of polymer (pDMA) only for the concentration range 0 to 10 µM. Emission spectra shown are acquired at a gate delay of 10 µs. There was no significant interaction between Eu^{3+} and pDMA judging by the identical luminescence spectra in the absence and presence of polymer.
